# Starch-Capped Silver Nanoparticles Impregnated into Propylamine-Substituted PVA Films with Improved Antibacterial and Mechanical Properties for Wound-Bandage Applications

**DOI:** 10.3390/polym12092112

**Published:** 2020-09-17

**Authors:** Mudassir Iqbal, Hadia Zafar, Azhar Mahmood, Muhammad Bilal Khan Niazi, Muhammad Waqar Aslam

**Affiliations:** 1 Department of Chemistry, School of Natural Sciences, National University of Sciences and Technology, Islamabad 44000, Pakistan; Hadia.zafar@yahoo.com (H.Z.); dr.azhar@sns.nust.edu.pk (A.M.); 2 School of Chemical and Materials Engineering, National University of Sciences and Technology, Islamabad 44000, Pakistan; m.b.k.niazi@scme.nust.edu.pk; 3 Department of Chemistry, University of Science and Technology Bannu, Bannu KPK 28100, Pakistan; waqar_aslam496@hotmail.com

**Keywords:** green synthesis, nanomaterials, antibacterial, polyvinyl alcohol, silver nanoparticles, wound bandages

## Abstract

This research endeavor aims to develop polyvinyl alcohol (PVA) based films capable of blends with silver nanoparticles (Ag–NPs) for improved antibacterial properties and good mechanical strength to widen its scope in the field of wound dressing and bandages. This study reports synthesis of propylamine-substituted PVA (PA–PVA), Ag–NPs via chemical and green methods (starch capping) and their blended films in various proportions. Employment of starch-capped Ag–NPs as nanofillers into PVA films has substantially improved the above-mentioned properties in the ensuing nanocomposites. Synthesis of PA–PVA, starch-capped Ag–NPs and blended films were well corroborated with UV/Vis spectroscopy, FTIR, NMR, XRD and SEM analysis. Synthesized Ag–NPs were of particle shape and have an average size 20 nm and 40 nm via green and chemical synthesis, respectively. The successful blending of Ag–NPs was yielded up to five weight per weight into PA–PVA film as beyond this self-agglomeration of Ag–NPs was observed. Antibacterial assay has shown good antimicrobial activities by five weight per weight Ag–NPs(G)-encapsulated into PA–PVA blended film, i.e., 13 mm zone inhibition against *Escherichia coli* and 11 mm zone inhibition against *Staphylococcus aureus*. Physical strength was measured in the terms of young’s modulus via tensile stress–strain curves of blended films. The five weight per weight Ag–NPs(G)/PA–PVA blend film showed maximum tensile strength 168.2 MPa while three weight per weight Ag–NPs(G)/PVA blend film showed highest values for ultimate strain 297.0%. Ag–NPs embedment into PA–PVA was resulted in strong and ductile film blend than pristine PA–PVA film due to an increase in hydrogen bonding. These good results of five weight per weight Ag–NPs(G)/PA–PVA product make it a potent candidate for wound dressing application in physically active body areas.

## 1. Introduction

Skin is the first line of our body defense against pathogenic microorganisms due to dense surface and corneous barrier texture [1,2]. Wound infection is the most important challenge for wound care management. Any breach in the skin by wound causes invasion of microorganisms into the sensitive underneath tissues that lead to wound infection. The advanced research on wound dressing development suggests that the ideal wound dressing should provide a wet environment to the wound, absorb exudates from the wound, should be highly biocompatible, having good antimicrobial activity for quick tissue regeneration and mechanical features to remain intact [3,4,5,6,7].

Hydrogels from polymers are gaining importance over the last few years for wound dressing because they have all the characteristics of an ideal wound dressing except lack the ability to form stable film due to weak mechanical properties. Problems can be overcome by functionalizing polymer with organic and inorganic agents [8,9,10]. Functionalized PVA is a potent candidate for dressing material owning to its non-toxicity, non-carcinogenicity, good biocompatibility and excellent degree of swelling in aqueous solutions [10,11]. Simple structure and geometric conformation of PVA ease its chemical modification on the secondary alcohol group. All the compounds that are able to react with the hydroxyl group can be used as potential cross-linking agents for PVA [1,12,13]. PVA has been reported to be derivatized by carbamation, esterification, etherification and click chemistry and the resulted products have unique properties and wide applications [14,15,16]. Bader et al. have derivatized PVA with 1, 2-epoxy-5-hexene as a cross-linker for hydrogels formation. The glass transition and melting temperatures of the modified PVA were 124–131 °C and 219–220 °C, respectively. The modifying agent 2-epoxy-5-hexene acted as cross-linker in the formation of hydrogels by photopolymerization of the monomers N-vinylpyrrolidone, 2-hydroxyethyl acrylate, N,N-dimethylacrylamide or acrylic acid, in the presence of 2-hydroxy-1-[4-(2-hydroxyethoxy)phenyl]-2-methyl- 1-propanone as photoinitiator. Based on the cytotoxicity tests, the hydrogels were claimed to be advantageous for biomedical applications [17]. C. Wang and coworkers were reported grafting of poly(4-vinyl pyridine) on PVA via radical polymerization in the presence of ceric ammonium sulfate as a radical initiator. This was reported to have good antifouling and antimicrobial properties than the non-grafted ones [18]. PVA has also been reported to modify with carbamate linkage via click reaction by using CDI as a coupling agent. Cross-linking was achieved by reaction between hydrazide modified PVA and aldehyde modified PVA. Resultant products had strong intermolecular attraction caused good tensile strength and mechanical properties. These hydrogels were reported to be stable for four months and low cytotoxicity [19]. Lim et al. have developed biosynthetic hydrogels Tyr–PVA by tyramine grafting through the succinyl groups initially attached to PVA in Dulbecco’s phosphate-buffered saline. Gel was formed by carbon–carbon bonds between phenol groups of the tyrosine of the gelatin and those of Tyr–PVA. Resultant material has good potential for bioengineering applications [20]. In another reported work, methacryloyl groups were grafted on PVA by reaction with GMA in DMSO. Hydrogels from GMA-modified PVA (GMA–PVA) and blends of GMA–PVA/chondroitin sulfate were prepared in the presence of N,N,N’,N’-tetramethylethylenediamine and sodium persulfate. Hydrogels from the blends showed greater swelling degrees that decreased with increasing degree of substitution. However, the mechanical properties of the hydrogels increased with an increasing degree of substitution. The biologic tests of the hydrogels revealed their nontoxicity for cell growth [21]. Zhao et al. have prepared a series of excellent hydrogels from PVA and carboxymethylated chitosan. The mechanical properties and equilibrium degree of swelling improved after adding CM–chitosan into PVA hydrogels. The gel fraction determined gravimetrically showed that a part of CM–chitosan was immobilized onto PVA hydrogel. The blend hydrogels exhibited good antibacterial activity against *Escherichia coli* [22]. PVA–PVP hydrogels containing Ag–NPs were prepared by Haijun and coworkers via repeated freezing-thawing treatment. It was found that a three-dimensional structure was formed during the process of freezing-thawing treatment and no serious aggregation of the Ag–NPs occurred. Good water absorption properties, the release of silver ions from the hydrogels, and the antibacterial effects of the hydrogels were observed against *E. coli* and *Staphylococcus aureus* [23]. A simple one-pot method for in situ synthesis of Ag–NPs within polyvinyl alcohol/gum acacia hydrogel matrix, by gamma radiation-induced cross-linking, is reported by Juby et al. The thermal stability was found to be more for the hydrogel loaded with Ag–NPs and also the percentage silver loading was found to increase with an increase in cross-linking density. These hydrogels have showed good antibacterial activity against Gram-negative bacterium *E. coli* [24].

Maribel et al., have prepared Ag–NPs by chemical reduction of silver nitrate with hydrazine hydrate and sodium citrate. Silver nanopowders have agglomerates of grains size 40–60 nm and face-centered-cubic nanoparticles of size 10–20 nm. The colloidal Ag–NPs inhibited the growth of the tested bacteria, including highly multi-resistant bacteria such as *S. aureus*, *E. coli* and *Pseudomonas aeruginosa* at very low inhibitory concentrations, i.e., 6.74 μg/mL [25]. Pseudospherical shaped and different sizes Ag–NPs (7, 29 and 89 nm) were synthesized by Martinez and co-workers using gallic acid via aqueous chemical reduction method. The antibacterial activity of these Ag–NPs was founded to decrease with an increase of the particle size [26]. Dongwei et al., have reported the synthesis of Ag–NPs and its impregnation into benign chitosan. Resultant products have demonstrated both fast and long-lasting antibacterial effectiveness against both Gram-positive and Gram-negative bacteria, comparable with the highly active precursor silver. This was due to dual actions viz., the bactericidal effect of Ag–NPs and cationic effects of chitosan [27]. Manikandan and co-workers have biosynthesized surface functionalized Ag–NPs by exposing aqueous silver ions to unripe fruits extract of *Solanum trilobatum* which exhibited good activity towards Gram-positive (*Streptococcus mutans*, *Enterococcus faecalis*) and Gram-negative (*E. coli*, *Klebsiella pneumoniae*) bacteria [28]. Cyclodextrins are cyclic oligosaccharides derived from starch which was used by Asli et al., as the reducing and stabilizing agent during the in situ synthesis of Ag–NPs via electrospinning of aqueous and DMF solutions using different Ag contents. This was revealed that the size of Ag–NPs depends upon solvent-type and Ag loading in nanofibers. These nanocomposites have shown significant inhibition against the growth of *E. coli* and *S. aureus* [29]. Marulasiddeshwara et al. reported the green synthesis of Lignin Capped Ag–NPs and their antibacterial potential. Results have indicated that spherical Ag–NPs grafted on lignin were highly crystalline with size 10–15 nm. This product has shown antibacterial activity against human pathogens *S. aureus* and *E. coli* while founded nontoxic to RBC cells taken together, hence, could be a promising therapeutic agent in the biomedical field [30]. Antimicrobial properties of Ag–NPs are well corroborated in literature [31,32,33,34]. However, its agglomeration in organic polymeric matrix limits its application in dressing polymeric materials. In the current study, Ag–NPs were capped with starch and embedded into PVA film which was modified with propylamine crosslinking for improved wound healing applications.

## 2. Materials and Methodology

### 2.1. Materials

All the materials viz. polyvinyl alcohol (Mw = 1500 amu, Sigma-Aldrich, Darmstadt, Germany), N-(*3-bromopropyl*)phthalimide (Sigma-Aldrich, Darmstadt, Germany), silver nitrate (BDH, England), Starch (Daejung, Korea), glycerin starch (Daejung, Korea), NaOH (Sigma-Aldrich, Darmstadt, Germany), sodium citrate (Sigma-Aldrich, Darmstadt, Germany), sodium borohydride (Sigma-Aldrich, Darmstadt, Germany) DMF (N,N-dimethylformamide) (Daejung, Korea), ethanol (Daejung, Korea), hydrazine (Daejung, Korea), isopropyl alcohol (Daejung, Korea) and chloroform (Daejung, Korea) were obtained from the commercial sources. All solvents including deionized water were of analytical grade and were used without further purification.

### 2.2. Synthesis of Silver Nanoparticles

Synthesis of Ag–NPs was carried out by two methods, i.e., chemical reduction method and green synthesis method (starch-capped Ag–NPs).

#### 2.2.1. Synthesis of Silver Nanoparticles via Chemical Reduction Method

As reported elsewhere, the chemical reduction method was used to synthesize Ag–NPs from its precursor AgNO_3_ [25]. Silver nitrate solution (2 mL, 2.5 mM) and citrate solution (2 mL, 2.5 mM) were mixed together in a reaction flask. Subsequently, NaOH solution (0.6 mL, 10 mM) was added into this. DI water was added to the makeup reaction solution volume up to 20 mL. The resultant solution was vigorously stirred until the slight clouding disappeared. Afterward, ice cold (0 °C) NaBH_4_ solution (0.6 mL, 10 mM) was added into this with stirring for 30 s. The light yellow color of reaction solution turned into dark yellow color confirming the formation of silver nanocolloids. The stirring was terminated right after the complete addition of NaBH_4_ solution to avoid agglomeration. Nanosize of the silver particles was also confirmed by performing UV-Vis absorption and SEM spectroscopy.

#### 2.2.2. Synthesis of Starch-Capped Silver Nanoparticles via Green Method

Starch-capped Ag–NPs were synthesized via green method as reported elsewhere [35,36]. Powdered starch (0.5 g) was added into the alkaline solution prepared by dissolving 0.25 g of NaOH in 5 mL DI water. The resultant solution was heated at 70 °C for about 10 min with stirring. In another flask, AgNO_3_ (0.6 g) was added into 2-mL DI water with stirring followed by the addition of 3 mL isopropyl alcohol for lowering boiling point to ease evaporation of solvent during the synthesis of Ag–NPs. This silver nitrate solution was added dropwise into previously prepared starch-alkali solution with stirring and heated at 70 °C for 30 min. The resultant yellowish-brownish colored solution has confirmed the synthesis of Ag–NPs. This solution was finally dried in a heating oven at 80 °C for 24 h which furnished powdered Ag–NPs.

### 2.3. Synthesis of Silver Nanoparticles Encapsulated PVA Films

PVA (1 g) was dissolved in 10 mL DI water at 80 °C with constant stirring for 4 h. Different concentrations (3% & 5% *w/w*) of starch-capped/chemically reduced Ag–NPs were mixed in DI water separately. The chemical cross-linking agent solution was made by adding 0.1 g glutaraldehyde and 0.01 mL HCl in 1 mL ethanol. Each prepared silver nanoparticle solutions were added separately to PVA solution at room temperature with constant stirring. Afterward, cross-linking agent solution and 0.4 g ethylene glycol were added to the mixtures with constant stirring and reaction mixtures were homogenized by sonication for 30 min. The resulting mixtures were cast onto the Petri dishes and left for drying at room temperature. After 24 h, the cured hydrogel membranes were removed from Petri dishes with the help of forceps and stored in airtight bags [37,38].

### 2.4. Synthesis of Propyl Amine-Substituted Poly Vinyl Alcohol Films

**Figure 1:**
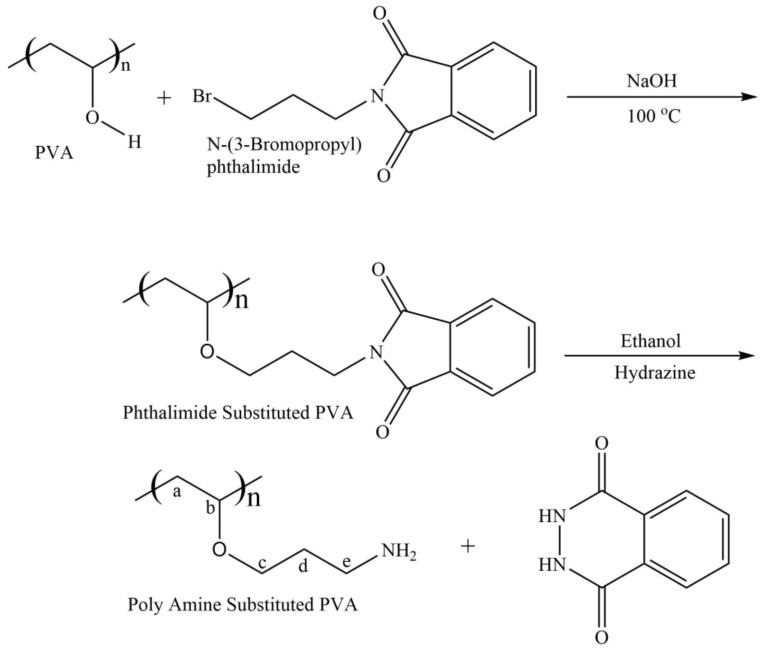
Chemical synthesis of propylamine-substituted polyvinyl alcohol (PVA).

Derivatized PVA was synthesized by a modified two-step reaction [39,40,41]. PVA (1 g) was added into a round bottom flask containing 50 mL DI water, heated at 80 °C for half an hour with stirring and left overnight at stirring to obtain a clear PVA solution. Subsequently, NaOH (0.92 g) was added into the PVA solution with stirring for 30 min. A solution containing 1 g of N-(*3*-*bromopropyl*) phthalimide into 40 mL DMF, was added in portions into the PVA solution with stirring. The resultant turbid reaction mixture was refluxed at 100 °C until clear homogenous solution was obtained. Afterwards, the reaction mixture was cooled to 0 °C in an ice bath and pH was neutralized by 10% HCl aqueous solution with constant stirring. The mixture kept undisturbed for 48 h which furnished gel-like material. The mixture was precipitated by the addition of 100 mL acetone while precipitates were filtered out and dried. In the next step de-protection of the phthalimide group was performed. Dried precipitates (0.5 g) were placed into a round bottom flask, hydrazine (2.5 g) was added along with ethanol (20 mL) and kept on reflux overnight. The schematic representation of the reaction is given in Figure 1.

### 2.5. Synthesis of Silver Nanoparticles Encapsulated Propyl Amine-Substituted PVA Films

Propyl amine PVA derivative (0.1 g) was dissolved into 10 mL boiling DI water by constant stirring for 4 h. The solution was allowed to cool down at room temperature. Previously prepared Ag nanocolloids were introduced into the derivatized PVA solution with gentle stirring for 30 min. Turning the dark yellow color of reaction mixture into a light yellow color indicated that encapsulation has occurred. The resulting colloidal solution was cast into the Petri dishes and dried in a vacuum oven for 24 h at 50 °C. Resultant hydrogel membranes were removed from Petri dishes with the help of forceps and stored in airtight bags [42,43,44]. Details of all prepared samples are mentioned in Table 1.

### 2.6. Instrumentations and Methods

Resultant synthesized materials were characterized by Fourier transform infrared spectroscopy (FTIR) using a Bruker platinum ATR model Alpha (Germany) spectrophotometer in the spectral range of 4000–750 cm−1 and X-ray diffraction analysis (XRD) using an X-ray diffractometer (D8 advance BRUKER) equipped with CuKα radiation source, having a wavelength of 0.154 nm and graphite monochromator in a 2θ range of 10°–80°. The morphology, size, and composition of synthesized materials were investigated via Scanning electron microscope (VEGA3 TESCAN) at an accelerating voltage of 20 kV. ^1^H-NMR was carried out at a Bruker AVANCE III 500 MHz spectrometer. Mechanical properties of prepared blends were studied according to ASTM D882 by using Shimadzu Trapezium–X Universal Testing Machine (AG-20RRKNXD Plus). Test was carried out using dumbbell shaped samples of 20 mm × 10 mm dimensions. The extension rate was kept 10 mm/min. five samples of each film were tested at 27 °C.

### 2.7. Antibacterial Activity

Antibacterial activities of all the prepared films were studied by the disc diffusion method against two microorganisms, i.e., Gram positive *S. aureus* and Gram negative *E. coli* bacteria. Gentamycin and PVA film were employed as positive and negative controls, respectively. Both strains of bacteria were cultivated in lysogeny broth and incubated at 37 °C for 24 h. Bacterial culture was uniformly spread on prepared agar plates. Sample discs of 6-mm diameter were placed on these plates with the help of a sterile forceps. Plates were incubated at 37 °C for 24 h and minimum inhibitory concentrations were measured [45,46,47]. All the experiments were done in triplicate and mean values with standard error are given in Table 2.

## 3. Results and Discussion

Aiming to synthesize a modified PVA with improved antimicrobial active wound dressing hydrogels, propylamine-substituted PVA was prepared by replacing the hydrogen of the PVA polymer (-OH) with propylamine (-CH_2_-CH_2_-CH_2_-NH_2_). The synthetic reaction of the derived PVA is shown in Figure 1. Propyl amine moiety was introduced in PVA by using N-(*3-bromopropyl*)phthalimide. PVA polymer has reacted with N-(*3-bromopropyl*)phthalimide to yield N-(3-propyl)phthalimide bonded PVA polymer. It was treated with monohydrated hydrazine to remove bonded phthalimide resulting propylamine-substituted PVA (PA–PVA).

### 3.1. FTIR Results

**Figure 2:**
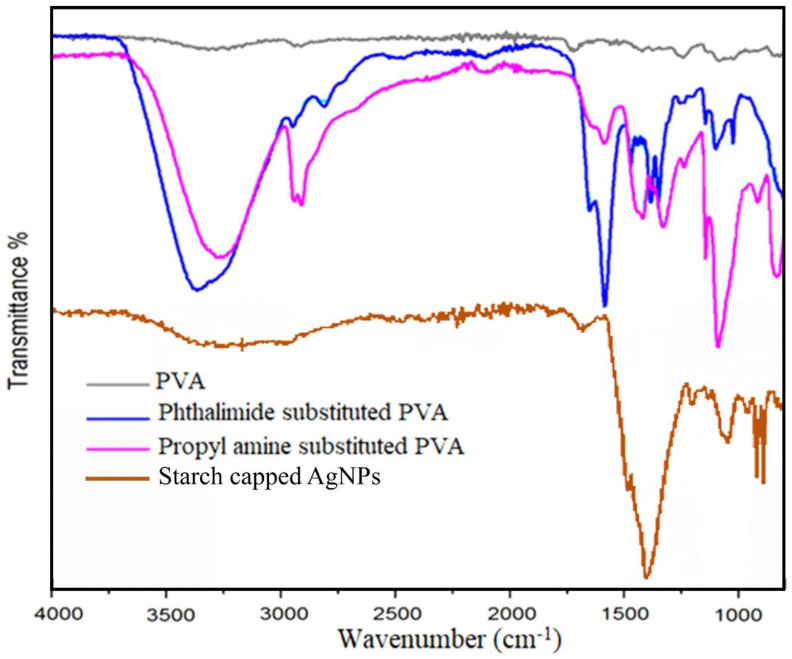
FTIR Spectra of PVA, phthalimide-substituted PVA, propylamine-substituted PVA and starch-capped Ag–NPs.

Along with TLC, the synthesis reaction of modified PVA was also monitored via FTIR spectroscopy. Indication of the bonding of N-(3-propyl)phthalimide with PVA and removal of the Phthalimide group was observed by vibrational studies using FTIR. In Figure 2 various vibrational bands have confirmed successful synthesis of N-propyl phthalimide -substituted PVA including C–H alkyl group stretching (2810–2940 cm^−1^), C=O (1650–1580 cm^−1^), CH_2_ sym band (1460 cm^−1^), CH_2_ asym band (1390 cm^−1^), C–N (1130 cm^−1^), C–O stretch (1080 cm^−1^) and =C–H banding (675–1000 cm^−1^) [48,49].

In the spectrum for propyl amine-substituted PVA (Figure 2), N–H stretch at 3500–3350 cm^−1^ was masked by the very strong O–H stretch at 3621–3315 cm^−1,^ but an overtone has emerged at 1590 cm^−1^ while the absence of vibrational bands of C=O indicated the removal of phthalimide. Furthermore, bands of C–H stretch (2810–2940 cm^−1^), CH_2_ sym band (1440 cm^−1^), CH_2_ asym band (1330 cm^−1^) and C–N stretch (1090 cm^−1^) have confirmed propyl amine substitution in PVA. Both spectra have shown that there was strong inter and intramolecular hydrogen bonding present because of O–H functionality and presence of associated water molecules [50,51]. FTIR spectrum of starch-capped Ag–NPs has exhibited shift in characteristic bands of starch like OH stretching has absorbed at 3143 cm^−1^, carbonyl stretching at 1634 cm^−1^, C–O–C symmetrical stretching at 1348 cm^−1^ and COH bending at 981 cm^−1^. These shifts in vibration bands have indicated interaction between functional groups of starch and Ag–NPs due to capping of Ag–NPs by starch [36,52,53].

### 3.2. NMR Analysis

^1^H-NMR spectroscopy has been widely used to derive the information about the local environment of the proton in the Polymer. Pristine PVA has characteristic CH_2_ doublet at chemical shift value δH 1.37 while the DMSO solvent resonates at δH 2.5 and water at δH 3.16. CH has demonstrated triplet at δH 3.8 due to shielding by OH while OH has furnished peak at δH 4.1 [54,55].

^1^H-NMR of the PA–PVA has depicted five peaks with different splitting and chemical shift values because of different chemical environments. PA–PVA resonances have included methylene proton δH 1.37 (2H_a_, doublet), methine proton δH 4.5 (1H_b_, triplet), methylene proton δH 4.2 (2H_c_, triplet), methylene proton δH 1.44 (2H_d_, nonet) and methylene proton δH 3.8 (2H_e_, triplet). Peak at δH 3.16 has shown that there was much water present in the sample. R-NH_2_ signal was observed between δH 1–2.5 which is overlapped by water and DMSO because of the small differences in chemical shift values and limited resolution power of spectrum. ^1^H-NMR of PA–PVA is in good agreement with the relevant FTIR results. Magnetic resonance of methylene protons (2H_c_) at downfield, i.e., δH 4.2 is justified by the close vicinity of highly electronegative oxygen atom [39,56]. Some peaks of the N-(*3-bromopropyl*)phthalimide were also found to be present at down fields in the spectrum around the DMSO peak. Methylene protons of the -CH_2_-bromo group have furnished peak at δH 3.43 in N-(*3-bromopropyl*)phthalimide [57,58].

### 3.3. XRD Analysis

X-ray diffraction pattern was analyzed to study the crystal structure of Ag–NPs precipitated by chemical and green methods [59,60]. For the Ag–NPs(C) synthesized by chemical method, characteristic peaks for face centered cubic (fcc) metallic silver were observed at 2theta 38°, 44°, 64° and 77° for the corresponding Bragg’s values (111), (200), (220) and (311), respectively (JCPDS card no. 04-0783).

**Figure 3:**
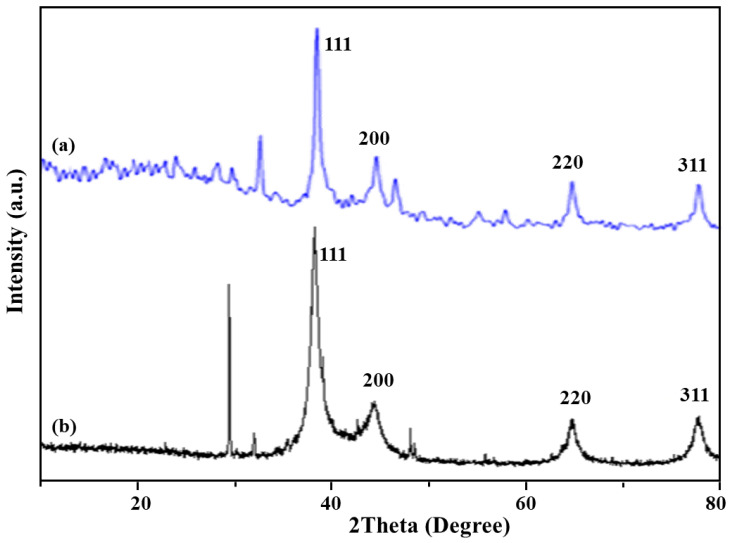
XRD of Ag–NPs prepared by: (**a**) chemical method; (**b**) green method.

In X-ray diffraction pattern for the green method, all characteristic Ag–NPs(G) peaks of the zero valent Ag–NPs have appeared in good agreement with the reported standard values of JCPDS No. 04-0783. Peak shapes have augmented the crystalline nature of Ag–NPs(G). On the basis of average crystallite sizes derived from XRD spectra using Scherrer’s equation, it was inferred that the starch employed in the green method has more reducing effect thus synthesized nanoparticles are of smaller crystallite size (9 nm) while by chemical method the synthesized nanoparticles are of larger crystallite size (20 nm). Some residual starch peaks were also observed in the XRD pattern of green synthesized nanoparticles (Figure 3).

### 3.4. UV-Vis Spectroscopy

**Figure 4:**
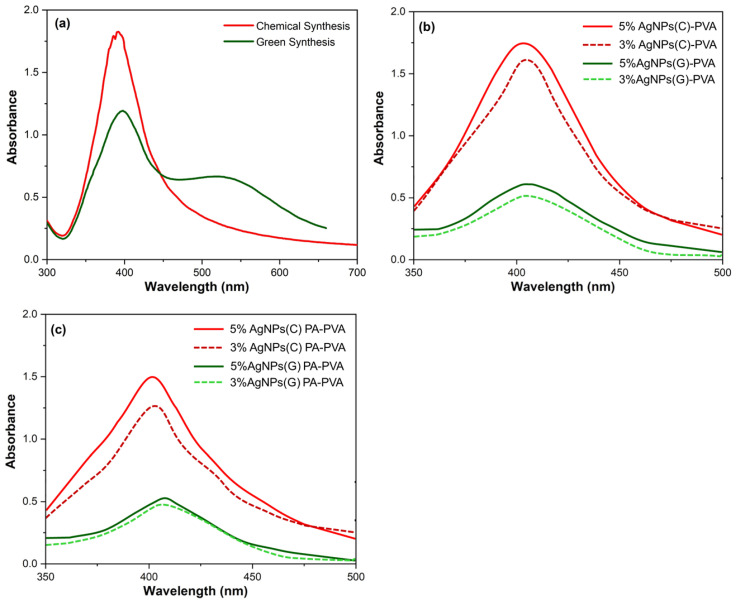
Surface plasmon resonance peaks of: (**a**) Ag–NPs (chemical and green synthesis); (**b**) Ag–NP-encapsulated PVA film; (**c**) Ag–NP-encapsulated PA–PVA.

Although color change helps in the visual assessment for the formation of Ag–NPs as already mentioned in the experimental section, but for the instrumental proof synthesized Ag–NPs were analyzed by UV-Vis spectrophotometer [61,62,63]. Uniformly suspended Ag–NPs synthesized by the green and chemical synthesis methods have furnished surface plasmon resonance peaks as shown in Figure 4. Appearance of the intense absorption peaks at 400 nm because of the collective excitation of all the free electrons is indicative of the successfully synthesized Ag–NPs.

Peak intensity has increased in the case of chemically synthesized Ag–NPs with a slight shift of peak to lower wavelength value. Surface plasmon resonance peaks intensity has indicated the formation of uniform Ag–NPs of smaller size distribution. Shift of the value from 1.8 to 1.2 has indicated that in the case of green synthesis, starch has influenced the size, reduction process and stabilization for the formation of Ag–NPs. Spectra for derivatized PVA encapsulated Ag–NPs(C) absorption is spread over a wide range, but the appearance of a curve around 400 nm has confirmed the presence of Ag–NPs. In the case of derivatized PA–PVA encapsulated Ag–NPs(G) the resultant dark color solution has absorption over a wide range. Peak positions in the case of derivatized PA–PVA have a slight difference of intensity indicating that both have different stabilization and reducing effect for the AgN’s synthesis. Moreover, the wavelength absorption at lower values means that synthesized Ag–NPs are of smaller size.

### 3.5. SEM Results

**Figure 5:**
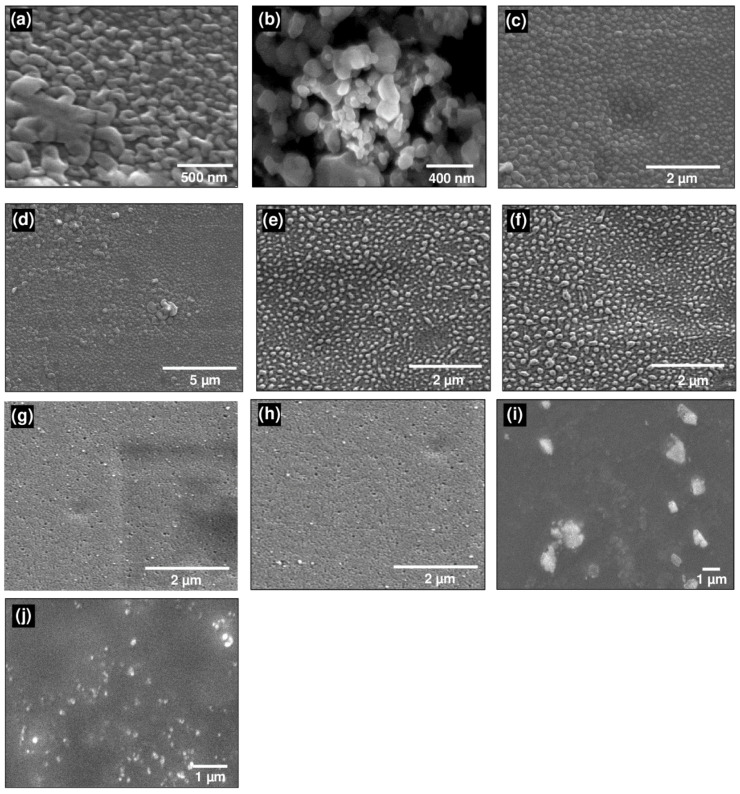
SEM micrographs of: (**a**) Ag–NPs (chemical synthesis); (**b**) Ag–NPs (green synthesis); (**c**) 3% Ag–NPs(C)–PVA film; (**d**) 5% Ag–NPs(C)–PVA film; (**e**) 3% Ag–NPs(G)–PVA film; (**f**) 5% Ag–NPs(G)–PVA film; (**g**) 3% Ag–NPs(C)-encapsulated PA–PVA; (**h**) 5% Ag–NPs(C)-encapsulated PA–PVA; (**i**) 3% Ag–NPs(G)-encapsulated PA–PVA; (**j**) 5% Ag–NPs(G)-encapsulated PA–PVA.

Scanning electron microscopy was employed for morphologic studies of Ag–NPs and Ag–NPs loaded PVA/PA–PVA films (Figure 5). SEM micrographs have depicted that synthesized nanoparticles have spherical shape while the size of particles were 19 nm and 40 nm for Ag–NPs synthesized by green and chemical methods, respectively. Green synthesis method was found to be more efficient than the chemical reduction method because starch capping effect caused difficulties in promoting lager size nanoparticles, so the growth of smaller size nanoparticles was favored. Surface morphology of the Ag–NPs loaded PVA films have shown that nanoparticles were evenly distributed in PVA films at 3% loading, but a slight clustering of nanoparticles was observed at higher concentration, i.e., 5% Ag–NPs [64,65,66].

SEM morphology of encapsulated Ag–NPs in PA–PVA film is depicted in Figure 5g–j. Particles synthesized in situ in PA–PVA film have a smaller size which was in line with the surface plasmon resonance peak results. This is due to the dense structure of the PA–PVA film which acted as the stabilizing as well as nucleation site for smaller sized Ag–NPs while the less dense PVA film provides enough space to grow relatively larger Ag–NPs.

### 3.6. Mechanical Testing

**Figure 6:**
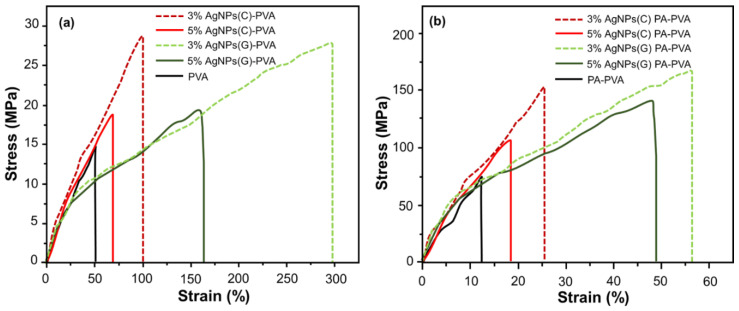
Stress–strain curves of: (**a**) Various Ag–NPs blended PVA films; (**b**) various Ag–NPs blended PA–PVA films.

Tensile stress–strain curves of Ag–NPs loaded PVA and substituted PVA films are shown in Figure 6. Different parameters obtained by performing mechanical testing were also tabulated in Table 2. These modified films have shown improved mechanical properties than pristine PVA film whose tensile strength was measured as 14.2 MPa. Ag–NPs loaded PVA and PA–PVA films were proven to be mechanically good. Tensile strength and strain percent at elongation and break point are depend on the Ag–NPs contents and the nature of polymers. Ag–NPs loading in PVA has resulted in strong and ductile film. PA–PVA film loaded with 5% Ag–NPs(G) has exhibited maximum tensile strength while 3% Ag–NPs(G) has demonstrated the highest values for ultimate strain percent thus the tougher material. For 3% loading of nanoparticles, the ultimate strain percent values were 98.8% for Ag–NPs(C) and 297.0% for Ag–NPs(G) which demonstrated 187% and 562% increment in the elongation at break values as compared to bare PVA films. Upon further increase of Ag–NPs (C & G) content in the films, i.e., 5% the ultimate strain% and tensile strength values were decreased. Tensile strength of 5% Ag–NPs(C)-PVA film was decreased to 18.5 MPa than 28.2 MPa of 3% Ag–NPs(C)-PVA film while for 5% Ag–NPs(G)-PVA film it decreased up to 19.3 MPa than 27.5 MPa for 3% Ag–NPs(G)-PVA film. This may be due to the fact that hydrogen bonding between the PVA and Ag–NPs decreased upon 5% Ag–NPs loading by agglomeration. Hydrogen bonding interaction was enhanced in the case of Ag–NPs(G) due to organo-compatible starch capping which led to higher values of tensile strength. Reinforcing effect of the Ag–NPs has increased the elongation values at the breaking point of polymer nanoparticle system. However, at elevated contents of Ag–NPs there was an agglomeration of the Ag–NPs in the polymer matrix which led to a decrease of hydrogen bonding and reinforcing effect thus causing failure of the polymer at lower tensile strength and ultimate strain% values.

Young’s modulus values of the PVA films impregnated with Ag–NPs were found between 26.1 MPa to 47.5 MPa. Ag–NPs(C)-PVA films with both 3% and 5% loading have shown higher values of modulus, i.e., 47.5 MPa and 35.5 MPa while Ag–NPs(G)-PVA films were failed at lower modulus values, i.e., 31.2 MPa and 26.1 MPa for 3% and 5% loading, respectively. Ag–NP-encapsulated PA–PVA film has shown a high modulus values of 1734.98 MPa.

PA–PVA films have exhibited relatively high Young’s modulus in the range of 104 to 168 MPa and tensile strength values range 1986.4 MPa to 2468.8 MPa. 5% Ag–NPs(G)-encapsulated PA–PVA film has shown maximum tensile strength among all samples, i.e., 168.2 MPa which is 227% higher than that measured for bare PA–PVA film (74.1 MPa). This composition has also exhibited maximum values of young modulus and% strain, i.e., 2468.8 MPa and 56.2%, respectively.

### 3.7. Antibacterial Assay

**Figure 7:**
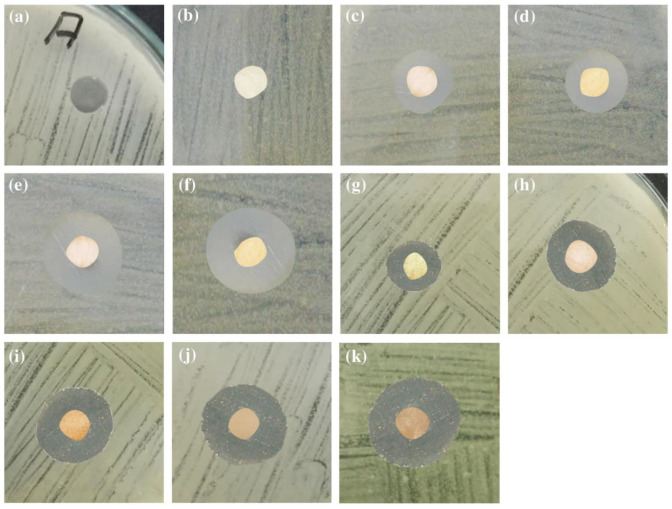
Antibacterial activities against *S. aureus* by: (**a**) blank; (**b**) pristine PVA; (**c**) 3% Ag–NPs(C)–PVA film; (**d**) 5% Ag–NPs(C)–PVA film; (**e**) 3% Ag–NPs(G)–PVA film; (**f**) 5% Ag–NPs(G)–PVA film; (**g**) pristine PA–PVA; (**h**) 3% Ag–NPs(C)-encapsulated PA–PVA; (**i**) 5% Ag–NPs(C)-encapsulated PA–PVA; (**j**) 3% Ag–NPs(G)-encapsulated PA–PVA; (**k**) 5% Ag–NPs(G)-encapsulated PA–PVA.

**Figure 8:**
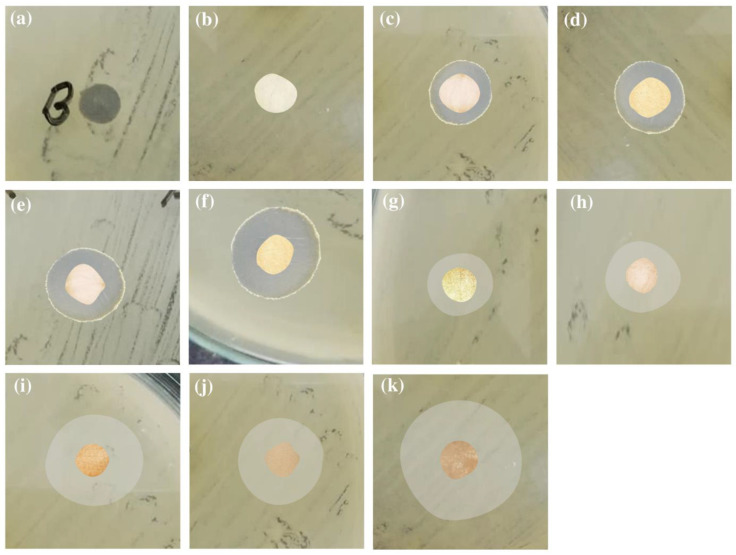
Antibacterial activities against *E. coli* by: (**a**) blank; (**b**) pristine PVA; (**c**) 3% Ag–NPs(C)–PVA film; (**d**) 5% Ag–NPs(C)–PVA film; (**e**) 3% Ag–NPs(G)–PVA film; (**f**) 5% Ag–NPs(G)–PVA film; (**g**) pristine PA–PVA; (**h**) 3% Ag–NPs(C)-encapsulated PA–PVA; (**i**) 5% Ag–NPs(C)-encapsulated PA–PVA; (**j**) 3% Ag–NPs(G)-encapsulated PA–PVA; (**k**) 5% Ag–NPs(G)-encapsulated PA–PVA.

All the prepared film samples were subjected to antibacterial assay against Gram positive *S. aureus* and Gram negative *E. coli* bacterial strains in the terms of inhibition zone measurement by disk diffusion method [67,68,69]. All as-prepared films have exhibited significant antibacterial activity than pristine PVA film (Figure 7 and Figure 8 and Table 3). The unsubstituted PVA films containing 3% and 5% Ag–NPs (green) concentration have demonstrated inhibition zones of 8 mm and 11 mm against *E. coli* while 9 mm and 10 mm against *S. aureus,* respectively. PVA films containing 3% and 5% chemically synthesized Ag–NPs have resulted 6 mm and 7 mm inhibition zone against *E. coli* while 7 mm and 7 mm against *S. aureus,* respectively. No inhibition zone was observed for bare PVA films.

Bare propyl amine-substituted PVA film has exhibited inhibition zone 5 mm against *E. coli* while 4 mm against *S. aureus,* respectively. The inhibition zone increased up to 9 mm and 13 mm against *E. coli* while 10 mm and 11 mm against *S. aureus* upon starch-capped Ag–NPs encapsulation into PA–PVA films.

## 4. Conclusions

Various substituted/unsubstituted PVA films impregnated with Ag–NPs prepared by chemical/green methods via starch capping were successfully synthesized and characterized by FTIR, NMR, UV-Vis spectroscopy, SEM and XRD while their mechanical attributes and antibacterial activities were also appraised. Synthesis of propylamine-substituted PVA was well corroborated in the FTIR spectrum where vibrational bands of OH group vanished, and new absorbance bands of amine were appeared above 3300 cm^−1^ corresponded to (O-CH_2_-CH_2_-CH_2_-NH_2_) substitution. Similarly, NMR spectra have depicted the disappearance of OH resonance at δ_H_ 4.1 while the emergence of amine peaks at δ_H_ 1–2.5. In UV-Vis Spectra, a sharp peak around 400 nm due to collective excitation of all the free electrons has indicated the successful formation of Ag–NPs. SEM micrographs have depicted particle shape morphology with sizes 19 nm for green and 40 nm for chemical synthesis of Ag–NPs. XRD pattern also confirmed the synthesis of Ag–NPs by characteristic intense peaks at 38 and 44 for metallic silver on 2θ reflections. All as-prepared films have exhibited significant antibacterial activity than pristine PVA film. The highest activity was demonstrated by 5% (*w/w*) Ag–NPs (G)-encapsulated into PA–PVA film. This has also shown maximum tensile strength among all samples, i.e., 168.2 MPa which was 227% higher than that measured for bare PA–PVA film. Thus, Ag–NP-encapsulated by PA–PVA film are good candidates for the wound dressing applications.

## Figures and Tables

**Table 1:** Details of all prepared samples.

Sample Code	Sample Details
Ag–NPs(C)	silver nano particles prepared by chemical method using NaBH_4_ as reducing agent
Ag–NPs(G)	silver nano particles prepared by green method via starch capping
Pristine PVA	unsubstituted poly vinyl alcohol film
3% Ag–NPs(C)–PVA film	3% *w/w* silver nano particles (prepared by chemical method) were loaded into unsubstituted poly vinyl alcohol film
5% Ag–NPs(C)–PVA film	5% *w/w* silver nano particles (prepared by chemical method) were loaded into unsubstituted poly vinyl alcohol film
3%Ag–NPs(G)–PVA film	3% *w/w* silver nano particles (prepared by green method) were loaded into unsubstituted poly vinyl alcohol film
5%Ag–NPs(G)–PVA film	5% *w/w* silver nano particles (prepared by green method) were loaded into unsubstituted poly vinyl alcohol film
Pristine PA–PVA	As-prepared propyl amine-substituted poly vinyl alcohol film
3% Ag–NPs(C)-encapsulated PA–PVA	3% *w/w* silver nano particles (prepared by chemical method) were encapsulated into propyl amine-substituted poly vinyl alcohol film
5% Ag–NPs(C)-encapsulated PA–PVA	5% *w/w* silver nano particles (prepared by chemical method) were encapsulated into propyl amine-substituted poly vinyl alcohol film
3% Ag–NPs(G)-encapsulated PA–PVA	3% *w/w* silver nano particles (prepared by green method) were encapsulated into propyl amine-substituted poly vinyl alcohol film
5% Ag–NPs(G)-encapsulated PA–PVA	5% *w/w* silver nano particles (prepared by green method) were encapsulated into propyl amine-substituted poly vinyl alcohol film

**Table 2:** Mechanical properties of various as-prepared samples.

Sample	Tensile Strength	Tensile Modulus	Ultimate Strain
	(MPa)	(MPa)	(%)
Pristine PVA	14.2 ± 3	21.8 ± 2	52.8 ± 5
3% Ag–NPs(C)–PVA film	28.2 ± 5	47.5 ± 3	98.8 ± 6
5% Ag–NPs(C)–PVA film	18.5 ± 6	35.5 ± 3	68.2 ± 3
3% Ag–NPs(G)–PVA film	27.5 ± 6	31.2 ± 3	297.0 ± 5
5% Ag–NPs(G)–PVA film	19.3 ± 7	26.1 ± 2	158.2 ± 2
Pristine PA–PVA	74.1 ± 5	1734.9 ± 3	13.2 ± 6
3% Ag–NPs(C)-encapsulated PA–PVA	152.6 ± 4	1986.4 ± 4	25.4 ± 5
5% Ag–NPs(C)-encapsulated PA–PVA	104 ± 6	2278.6 ± 6	18.7 ± 7
3% Ag–NPs(G)-encapsulated PA–PVA	147.5 ± 4	2037.2 ± 5	48.9 ± 7
5% Ag–NPs(G)-encapsulated PA–PVA	168.2 ± 5	2468.8 ± 7	56.2 ± 7

**Table 3:** Antibacterial activities of various as-prepared samples.

Sample	Inhibition Zone (mm)	
	*Escherichia coli*	*Staphylococcus aureus*
Pristine PVA	0	0
3% Ag–NPs(C)–PVA film	6 ± 0.1	7 ± 0.3
5% Ag–NPs(C)–PVA film	7 ± 0.15	7 ± 0.2
3% Ag–NPs(G)–PVA film	8 ± 0.4	9 ± 0.25
5% Ag–NPs(G)–PVA film	11 ± 0.3	10 ± 0.3
Pristine PA–PVA	5 ± 0.2	4 ± 0.3
3% Ag–NPs(C)-encapsulated PA–PVA	8 ± 0.25	8 ± 0.15
5% Ag–NPs(C)-encapsulated PA–PVA	10 ± 0.3	9 ± 0.2
3% Ag–NPs(G)-encapsulated PA–PVA	9 ± 0.15	10 ± 0.1
5% Ag–NPs(G)-encapsulated PA–PVA	13 ± 0.1	11 ± 0.2
